# Impact of different levels of lactose and total solids of the liquid diet on calf performance, health, and blood metabolites

**DOI:** 10.5713/ab.23.0225

**Published:** 2024-01-20

**Authors:** Gercino Ferreira Virgínio, Cécile Anna Jeanne Duranton, Marilia Ribeiro de Paula, Carla Maris Machado Bittar

**Affiliations:** 1 Minas Gerais Agricultural Research Agency, Experimental Field of Montes Claros, Montes Claros, Minas Gerais, 39404-128, Brazil; 2 Department of Animal Science, Luiz de Queiroz College of Agriculture, University of São Paulo, Piracicaba, 13418–260, Brazil

**Keywords:** Albumin, Calf, Fecal Score, Growth, Milk Replacer

## Abstract

**Objective:**

This study aimed to evaluate the effect of feeding milk replacer (MR) with varying levels of lactose and the increased supply of total solids (from 750 to 960 g/d) on performance, blood metabolites, and health of Holstein male calves during the preweaning period.

**Methods:**

Forty newborn Holstein calves (10 per treatment) were blocked according to birth weight and date of birth and distributed in a randomized block design to different liquid diets: Whole milk powder (WMP) diluted to 125 g/L solids; MR with 48% lactose (48L), diluted to 125 g/L solids; MR with 53% lactose (53L), diluted to 125 g/L solids; 53L MR corrected to 160 g/L solids (16TS) by the inclusion of a solid corrector. Calves were individually housed in wood hutches, fed 6 L/d of the liquid diet, and had free water and starter concentrate access. The study lasted 56 days.

**Results:**

Liquid diet intake was higher for calves fed 16TS than for other treatments. Calves fed 16TS presented higher protein and fat intake, followed by those fed WMP and the 48L or 53L MRs. Lactose intake was higher for 16TS-fed calves, followed by 53L, 48L, and WMP-fed calves. Starter and total dry matter intake did not differ among liquid diets. The average daily gain was higher for 16TS than 48L-fed calves, with the other treatments being intermediary. The lowest feed efficiency was observed for calves fed 48L. No effects on health were observed, as well as on selected blood metabolites, except for albumin concentration, which was higher for calves fed 16TS and WMP.

**Conclusion:**

Higher total solids content (160 g/L) in MR increases nutrient intake and consequently improves the performance of dairy calves. Feeding MRs with levels of lactose up to 53% of the DM had no deleterious effect on the performance or health of the calves.

## INTRODUCTION

The pre-weaning phase is a period of great sanitary and physiological challenges. The quality of liquid diets and feeding rate explain most of the performance [1]. The National Academies of Sciences, Engineering, and Medicine (NASEM) [2] has recommended that calves are liquid-fed according to high (>900 g/d) or moderate (600 to 900 g/d) feeding rates to improve energy supply for the immune system and growth rate. Several studies summarized by Soberon et al [3] have demonstrated that higher growth rates, either by the high liquid volume or starter intake, lead to greater milk yield in first lactation. For this reason, traditional milk feeding systems (3.8 to 4.7 L/d) tend to be replaced by feeding larger volumes of liquid diets (>7.6 L/d) [4] or higher-density liquid diets containing more solids (i.e., lactose, fat, and protein).

The use of milk replacers (MR) has several benefits, such as the independence of the milking schedule to feed calves, consistent liquid diet composition [5], and the possibility of offering a high-solids liquid diet. Feeding more solids in the liquid diet increases dry matter (DM) intake and weight gain [6] without challenging calves to consume higher volumes, especially when two meals are adopted, or calves are very young.

Nonetheless, the high costs have caused the dairy industry to search for ingredients with a high biological value that can replace current ingredients in formulations without negatively impacting animal health and performance. Consequently, nutritional levels are sometimes far different from what is found in whole milk (WM) [7].

On average, milk components of Holstein cows comprise 29.4% fat, 25.2% crude protein (CP), and 38.2% lactose on a DM basis, considering 125 g/L solids [8]. However, commercial MRs typically contain higher levels of lactose [9], lower levels of fat [10], and similar levels of protein [7]. According to Quigley et al [11], calves present the highest DM, N, and fat digestibility of MR or WM at about 30 d of age. Nevertheless, authors have observed a large range of nutrient digestibility in their meta-analysis, possibly due to nutrient levels and sources used in MR formulations. Such formulations can result in different animal performances and health.

The high levels of lactose in MR have been associated with scouring when fed in excess (~17.2 g/kg body weight) [12]. In addition, a lowered response to circulating glucose [13] may be associated with insulin insensitivity, and this can lead to a high level of fat catabolism and promote ketone body formation [14], impacting animal performance and health. The *ad libitum* intake of high-lactose MR was greater than high-fat MR, possibly indicating that calves displayed fewer signs of satiety [15].

Comparing similar solids feeding levels, the contribution of energy (Mcal) from MR with higher lactose and lower fat results in lower metabolizable energy (ME) intake than WM [10]. Because of that, increasing the supply of total solids in the liquid diet above 12.5% when feeding MR may increase the performance of dairy calves [6], close to what is observed with milk feeding. Feeding more solids may be done by changing the dilution rate of a particular MR or adding a corrector, which usually presents higher protein levels, in milk or a 12.5% diluted MR. However, solids higher than 15% may increase the occurrence of abomasal bloat [16] because of the high liquid diet osmolality, mainly when lactose is increased.

Several studies comparing MR formulations focus on protein levels and sources [2]. However, comparison of liquid diets with different lactose contents, especially when comparing with higher total solids contents is not common. Studies have been conducted to understand possibility of replacing lactose for glucose [9] or fat [10], and also looked at the effect of high lactose with intestinal health [12]. In this context there is a need of information of high lactose levels in modern MR formulation on dairy calves raising.

Differences in MR lactose content and total solids intake may affect calves' performance, metabolism, and health in the preweaning period. Thus, this study aimed to evaluate the impact of feeding MR with different levels of lactose and increasing the supply of total solids (from 750 to 960 g/d) on performance, blood metabolites, and health of Holstein male calves in the preweaning period.

## MATERIALS AND METHODS

### Animal ethics

All animal procedures were performed in accordance with the relevant guidelines and regulations, and were approved following the ethics by the Institutional Animal Care and Use Committee (Protocol no. 2013-8).

### Animals and facilities

The study was conducted at the experimental calf facilities of the Animal Science Department at "Luiz de Queiroz" College of Agriculture, University of São Paulo, Piracicaba, SP, Brazil.

Forty newborn male Holstein calves (36.2±5.0 kg of birth weight) from a commercial farm (≈ 100 km) were used in this study. Immediately after birth, all calves were separated from the dam, weighted, and fed 2 L of high-quality colostrum (>50 g IgG/L) within 2 h after birth and a second meal of 2 L the following 6 h later [17]. On the second day of life, calves were fed 4 L/d of maternal transition milk and transported to the experimental calf facilities. Calves were distributed in a randomized block design and blocked according to birth weight and date of birth (10 blocks, 10 calves per treatment). After the first colostrum feeding to ensure adequate passive transfer, a blood sample was collected from the jugular vein 48 h using a vacuum tube containing clot activator (VACUETTE do Brasil, Campinas, SP, Brazil). Only animals with serum protein above 5.5 g/dL were enrolled in the study.

### Management and feeding

All calves were housed individually in wood shelters (1.35 m in height, 1 m in width, and 1.45 m in depth), with free access to water and a commercial starter concentrate (Table 1; Agroceres Multimix, Rio Claro, Brazil). All animals were bucket-fed with 6 L/d, divided into two meals (7 h and 17 h), of one of the four evaluated liquid diets, during 56 d: WMP, whole milk powder diluted to 125 g/L solids; 48L, a commercial MR with moderate lactose (485 g DM/kg) diluted to 125 g/L solids; 53L, a commercial MR with high lactose (533 g DM/kg) diluted to 125 g/L solids; 16TS, 53L-treatment MR added 35 g/L of a solids corrector to achieve 160 g/L of solid content (Table 1). The solids corrector contained 255 g/kg CP, 163 g/kg ether extract (EE), 483 g/kg lactose, and 59 g/kg of ash on a DM basis. The solids corrector was used not only to increase the solids content of the liquid diet offered, but also to provide a higher content of nutrients (lactose, fat and protein) to be ingested by the calves. The MR was diluted in drinking water heated to 45°C, according to the manufacturer's instructions, and provided to the animals at 37°C to 38°C.

Potable water and starter concentrate were available throughout the day to all calves for *ad libitum* consumption preweaning. Starter concentrate was supplied each morning after milk feeding and was available until the next morning when leftovers were weighed to calculate daily intake. The leftover liquid diet was measured, and intake was also recorded daily.

### Feed analysis

Samples of MR and starter were collected weekly for later analysis [18]. The dry matter was measured by drying at 100°C in a forced-air oven for 24 h and ashes by furnace incineration at 550°C for 4 h (method 942.05). The EE was determined using petroleum ether (method 920.39), with acidification with glacial acetic acid for the MR samples. Crude protein was analyzed using the Dumas method [19] with an N analyzer (FP-528, Leco, St. Joseph, MI, USA). Lactose concentrations were analyzed according to Feitosa-Teles et al [20]. Free-ash neutral detergent fiber (NDF) was determined according to Van Soest et al [21] and acid detergent fiber according to Goering and Van Soest [22], using sodium sulfite and thermo- stable amylase. The non-fiber carbohydrate (NFC) of the starter and MR were estimated according to the following equation: NFC (%) = 100%−(% NDF+% CP+% fat+% ash), according to Mertens [23]. The metabolic energy of the liquid diets was calculated according to NASEM [2] by multiplying gross energy (GE Mcal/kg DM = ([fat×9.4]+[protein×5.7]+[100−protein−fat−ash×4]) /100) by 0.93 for the whole milk powder and by 0.91 for the MRs.

### Performance, body measurements, and health

All calves were weighed weekly before the morning feeding on a mechanical scale (ICS-300; Coimma Ltda., Dracena, SP, Brazil). The withers height and hip width were measured using a stick with a cm-scale (ruler), and the heart girth using a measuring tape. Feed efficiency was calculated as the gain-to-feed ratio. Every morning, the fecal score was monitored, as described by Larson et al [24], regarding the fluidity of feces: i) normal and firm, ii) loose but with a generally healthy aspect, iii) very loose, not liquid separation, and iv) watery. Calves with a score ≥ of 3 received oral rehydration solution (5 g of NaCl, 25 g of dextrose, and 10 g of bicarbonate/L) 2 h after morning feeding, with a bottle until the fecal score returned to normal (≤2). Health problems were monitored and treated according to veterinary recommendations. All treatments were registered.

### Blood sampling and analysis of metabolites

Blood samples were collected weekly, 2 h after morning feeding, to analyze the biochemical and metabolic profiles during the preweaning phase. The samples were collected by jugular vein puncture in two vacuolized tubes. Plasma was isolated from a tube containing sodium fluoride as an antiglycolytic plus potassium ethylenediaminetetraacetic acid as an anticoagulant, and the serum was obtained from a tube containing a clot activator. An aliquot of blood was used to analyze the capillary hematocrit, which was centrifuged in a microhematocrit centrifuge (SPIN 1000, MICROSPIN, USA), at 12,000×g, for 10 minutes. After centrifugation, capillaries were read using a hematocrit ruler. Samples were centrifuged at 2,000× g for 20 min at 4°C to obtain plasma or serum and were stored at −20°C for subsequent analysis. Specific commercial enzymatic kits (Labtest Diagnóstica S.A., Lagoa Santa, Brazil) were used to analyze plasma glucose (ref. 85), total serum protein (TSP; ref. 99), serum albumin (ref.^19^–1/250). All metabolites were measured in an automatic biochemistry system (SBA—200; CELM, Barueri, SP, Brazil).

### Statistical analysis

Statistical analyses were performed using the MIXED procedure of the SAS statistical package. All data were tested for normal distribution by the Shapiro-Wilk test and homogeneity of the variances using the Levene test. For the variables feed, and nutrients intake, average daily gain, body weight, and measures gain, feed efficiency, fecal score, and blood metabolites, the analysis was performed as repeated measures over time (weeks of age), with the following statistical model: Y_ijk_ = μ+D_i_+b_j_+e_ij_+A_k_+(bA)_jk_+(DA)_ik_+e_ijk_. Where, μ = general average; D_i_ = fixed effect of diet; b_j_ = random block effect; e_ji_ = residual error; A_k_ = fixed age effect; (bA)_jk_ = random effect of block × age interaction; (DA)_ik_ = fixed effect of the diet × age interaction, and e_ijk_ = residual error B. The model included treatment effects, week (age of calves), and the interaction between treatment and week as fixed effects. The block effect was included in the model as a random effect. The subject of the repeated measures was animal within the treatment. The covariance matrices "compound symmetry, heterogeneous compound symmetry, autoregressive, autoregressive heterogeneous, unstructured, banded, ante-dependence, variance components, toeplitz, and heterogeneous toeplitz" were tested and defined according to the lowest value obtained for "Akaike's Information Criterion Corrected" (AICC) and the subject of the repeated measures used was animal (treatment). The non-repeating data (initial weight, weaning weight) were evaluated using the following statistical model: Y_ij_ = μ+D_i_+b_j_+e_ij_, where μ = general mean; D_i_ = fixed effect of diet; b_j_ = random block effect; and e_ij_ = residual error. The block was included as a random effect. For all response variables, the means were obtained using the LSMEANS command. The comparisons among the treatments were performed by the Tukey test when there was significance in the analysis of variance. Significance was declared when p≤0.05, and a trend was declared at 0.05≤ p≤0.10.

## RESULTS

### Growth performance

**Figure 1:**
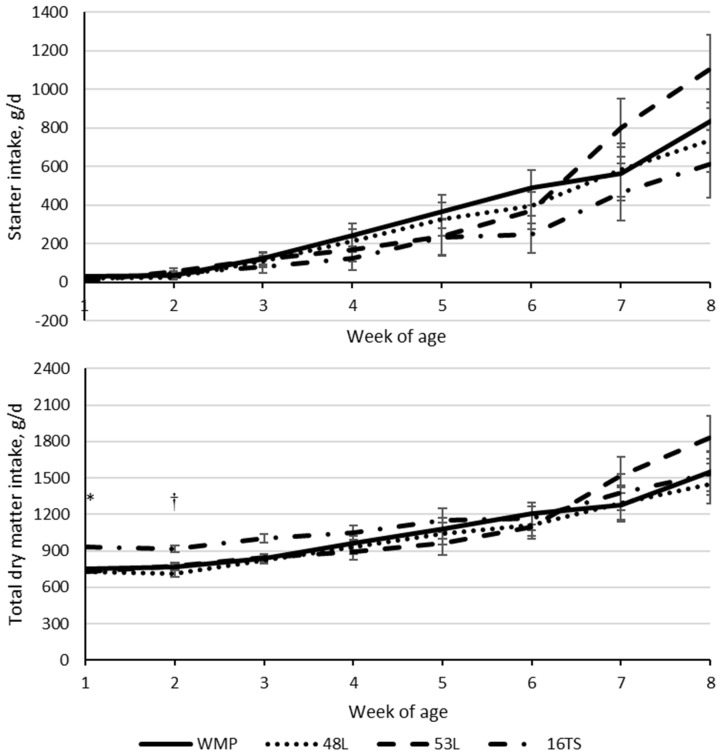
Starter and total dry matter intake (g/d) according to age of calves fed with different liquid diets. WMP, whole milk powder diluted to 125 g/L solids; 48L, milk replacer with moderate lactose (485 g dry matter/kg) diluted to 125 g/L solids; 53L, milk replacer with high lactose (533 g dry matter/kg) diluted to 125 g/L solids; 16TS, the 53L milk replacer corrected to 160 g/L total solids by the inclusion of a corrector. * 16TS differs from all the others with p<0.05. ^†^ 16TS is higher than 48L with p<0.05.

**Figure 2:**
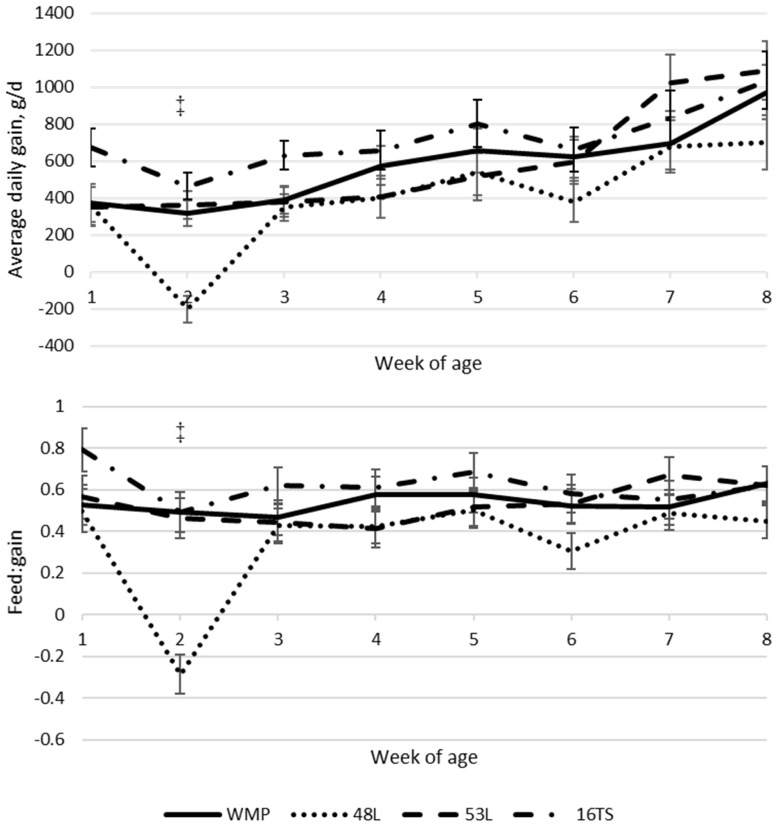
Average daily gain (g/d) and feed:gain according to age of calves fed with different liquid diets. WMP, whole milk powder diluted to 125 g/L solids; 48L, milk replacer with moderate lactose (485 g dry matter/kg) diluted to 125 g/L solids; 53L, milk replacer with high lactose (533 g dry matter/kg) diluted to 125 g/L solids; 16TS, 53L milk replacer corrected to 160 g/L total solids by the inclusion of a corrector. ‡ 48L differs from all the others with p<0.01.

Intakes of starter (p<0.001), liquid diet intake (p = 0.002), and total dry matter (TDMI; p<0.001) increased with age (Table 2). The starter intake was influenced by the interaction of age and treatment (p = 0.030), while TDMI showed a trend (p = 0.056) for the same effect (Table 2). Calves fed the high-solids liquid diet (16TS) presented the highest TDMI at weeks 1 and 2 (Figure 1). Also, the 16TS resulted in the highest dry matter intake in the whole period from the liquid diet (p<0.001), and the highest lactose (p<0.001), protein (p<0.001), and fat (p<0.001) intake during the whole period (Table 2). Fat and protein intake were intermediary for calves fed WMP and lower for 48L and 53L (p<0.001) (Table 2). However, lactose intake was lower for WMP, followed by the low lactose MR and the high lactose MR, with the highest intake observed for 16TS-fed calves (p<0.001; Table 2). The birth weight did not vary among treatments. However, calves fed a 16TS liquid diet tended to be heavier at weaning when compared to the 48L-fed group (76.6 vs 59.1 kg; p = 0.054; Table 2). This difference was significant when the average daily gain (ADG) was analyzed, with an increase of 320.8 g/d, i.e., 80% higher, for calves fed 16TS compared to those fed 48L (p = 0.0451; Table 2). In addition, animals in the 48L group had lower feed efficiency (0.35) than the WMP and 16TS groups (0.54 and 0.62, respectively), with the 53L being the intermediary. Both variables increased with age (p<0.001) and showed an age × treatment interaction effect, where the 48L group had the lowest ADG and feed efficiency (FE) values compared to the other treatments at the second week of life (Figure 2). No differences were observed in the following weeks (Table 2). No treatment, age, or interaction were observed in body weight or measurements (Table 2). The hip-width (p = 0.010) and hearth-girth (p = 0.024) measures gain increased with age (p<0.03), but only hip-width gain was different between 16TS and 48L treatments (0.77 and 0.48 cm/wk, respectively; p = 0.010), with the other liquid diets being intermediary (Table 2).

### Blood and health parameters

Selected blood parameters were affected by the age of the animals (p<0.01), with an increasing percentage in hematocrit and albumin concentration, while glucose and total protein concentration decreased over time (Table 3). The hematocrit values were higher for the 16TS-fed calves than the 48L group (22.9% vs 19.0%; p = 0.024). The different liquid diets also affected albumin concentration, with significantly lower values for the 48L and 53L (2.53 and 2.63 g/dL, respectively) than the other two (p<0.001). An interaction trend of treatment and age effect was observed for glucose concentration but with no differences among liquid diets within a particular week. The different liquid diets did not affect the fecal score, days with diarrhea, and days medicated or on electrolytes (Table 4). There was an age effect for fecal scores, with higher scores from weeks 1 to 4 but decreased afterward.

## DISCUSSION

Supplying sufficient nutrients by liquid diet feeding, whether WM or MR, is essential to improved performance and welfare of dairy calves during the preweaning phase. In our study, the higher performance of calves fed the 16TS liquid diet results from the higher observed nutrient intake from the liquid diet (lactose, fat, and protein), achieved by the increased solids concentration (160 g/L) by the addition of a corrector with high protein and fat composition. Our study observed a higher solid content of the liquid diet than supplying values close to the conventional (125 g/kg), consistent with other findings [25,26].

The protein concentration in the MR is a significant factor in determining growth in dairy calves, with several studies showing positive effects of increased protein content [27–30]. This effect mainly occurs when calves are fed higher liquid diet volumes and total solids [2,31]. However, this nutrient alone does not seem to guarantee the success of a feeding program because, in addition to the higher protein level, the use of more digestible protein sources is necessary [7,32]. The protein intake was the highest for calves fed 16TS, resulting in an ADG of 721 g/d. The results are consistent with Davis and Drackley [1], who observed that calves consuming 200 g CP/d achieved similar ADG as the current study. Calves fed WMP consumed lower CP than calves fed 16TS. However, ADG and FE were not statistically different, suggesting the importance of protein biological values in MR.

Most of the performance differences can be explained by liquid diet intake since there was no effect of liquid diet composition on starter intake, as observed by others studies [15,27,33]. Data shows that liquid diet intake represents about 70% of the total DMI. Together with the fact that digestibility of nutrients and nutrient use efficiency is higher for liquid diet than for solid diet nutrients, suggests that the nutrient intake from liquid diet dives growth rates. There was a difference of about 15% in the starter intake when 48L is compared to 53L, and an even higher difference when 16TS is compared to any treatment. However, those differences were not significant, because of the high variation in the solid diet feed intake. Nevertheless, a decrease in starter intake is expected as the DMI of liquid diet increase [34]. However, Cowles et al [35] reported higher starter intake when the protein content in the MR was reduced from 28% to 20%. Our study did not observe such an effect, even with two treatments, 48L, and 53L, with approximately 210 g/kg CP, probably because calves were fed using a moderate feeding program (6 L/d). The CP:ME (g/Mcal) was higher for WMP, but using a corrector efficiently increased this ratio in the MR. Besides that, ADG may be more positively related to higher total solids intake, as observed in our data and other studies [6,29,33].

Increased lactose content in MR, compared to WMP, did not affect fecal scores, diarrhea, days with diarrhea or medicated, days on electrolytes, or abomasal occurrence, similar to findings by Hof [12] and Hugi et al [36]. Also, no effects were observed on starter intake, as Wilms et al [15] observed, even though fat content was similar among MR but lower than WMP. Lactose is an important energy source for dairy calves, as it is almost completely oxidized [37]. However, instead of fattening and altering the gain composition, it is used to fuel protein synthesis [38]. Even though there may be a limit to lactose inclusion in MR formulation, extra energy will allow more body protein deposition [2]. However, our data do not show differences in withers height and heart girth to confirm that calves presented improved skeletal-muscle growth.

Recently, several papers were published on replacing lactose by fat in MR with a higher lactose content ranging from 443 to 553 g/kg [10,15,38]. These authors found no effect of high lactose diets on fecal scores or health problems. Except for Berends et al [10], who observed increased lung problems in calves fed the high lactose MR. These results suggest that even with lactose content as high as 53.3% in the present study and 55.3% in the study of Tikofsky et al [38], this supposed effect may occur only when lactose content is above published levels. On the other hand, Hugi et al [36] observed a lower fecal consistency when comparing MRs with 29% or 42.3% of lactose; however, with no effect on performance. More studies are needed to understand effects of high lactose and its relationship with other nutrients in the MR formulation and feeding level.

Feeding more solids in the liquid diet is also related to more fluid feces in dairy calves due to increased osmolality, resulting in decreased water absorption in the gut and causing osmotic diarrhea [25,26]. However, our results agree with Azevedo et al [6], who observed no differences in fecal score or days with diarrhea when calves were fed greater total solids (204 g/L).

Plasma glucose and serum protein were not affected by the different liquid diets, although there were differences in intake of lactose and CP of around 140 g and 52, respectively. The high lactose intake did not affect plasma glucose, even though literature has reported effects on glucose metabolism in young calves [13]. All calves were weekly sampled 2 h after liquid diet feeding when there is the glucose peak for MR-fed calves, but not for WM-fed calves, which is observed an hour later [39]. However, albumin blood concentrations, a negative acute phase protein with observed decreases in concentration during periods of inflammation [15], were affected by a liquid diet. Our data suggest a lower inflammation status for calves fed 48L and 53L; however, differences should be at least 25% indicative of an acute phase response [40]. Among other roles, albumin can contribute up to 75% of the blood plasma osmolality [41] and presents variable concentrations according to other blood proteins due to post-prandial effects. The lower albumin concentration for calves fed 48L and 53L may be more related to a greater dilution effect on the plasma of calves that present a lower intake of nutrients, as observed by Schäff et al [42]. Klinkon and Ježek [41] reported an average albumin concentration of 2.75 g/dL for pre-weaned dairy calves, indicating that 48L and 53L treatments were below the recommended value.

## CONCLUSION

The ingestion of a higher nutrient content by increasing the total solids (160 g DM/L) in the MR fed to the calves was more effective at improving performance than feeding whole milk (125 g DM/L). In our study, feeding MRs with levels of lactose around 530 g/kg of DM had no deleterious effect on the performance or health of the calves.

## Figures and Tables

**Table 1:** Chemical composition of starter and the different liquid diets

Chemical composition (g/kg)	Starter feed	Treatments^1)^			

		WMP	48L	53L	16TS
DM	892	955	956	963	965
Crude protein	244	264	209	208	218
Ether extract	34	256	154	154	156
Lactose	NA^2)^	408	485	533	522
Non-fiber carbohydrate	466	-	-	-	-
NDF	179	00	5.3	3.5	2.8
ADF	57	-	-	-	-
Ashes	77.3	59	75	45	44.7
ME (Mcal/kg DM)^3)^	-	5.13	4.40	4.51	4.54
CP:ME (g/Mcal)	-	51.3	47.5	46.0	48.0

DM, dry matter; NDF, neutral detergent fiber; ADF, acid detergent fiber; ME, metabolizable energy; CP, crude protein.

1) WMP, whole milk powder; 48L, milk replacer with moderate lactose (485 g DM/kg); 53L, milk replacer with high lactose (533 g DM/kg); 16TS, 53L milk replacer corrected to 160 g/L total solids by the inclusion of a corrector.

2) Not analyzed.

3) Calculated according to NASEM (2021) by multiplying gross energy by 0.93 for the whole milk powder and by 0.91 for the milk replacers.

**Table 2:** Intake and performance of calves fed with different liquid diets

Items	Treatments^1)^				SEM	p-value^2)^		
	
	WMP	48L	53L	16TS		LD	A	LD×A
Intake (g DM/d)								
Starter concentrate	335.7	302.1	358.9	227.7	69.95	0.599	<0.001	0.030
Liquid diet	716.7^bc^	709.8^c^	722.2^b^	914.3^a^	2.90	<0.001	0.002	0.134
Lactose^3)^	303.62^d^	344.88^c^	384.9^b^	446.84^a^	7.95	<0.001	0.278	0.993
Protein^3)^	189.2^b^	148.3^c^	150.0^c^	200.9^a^	0.51	<0.001	0.007	0.493
Fat^3)^	183.5^b^	109.3^c^	111.4^c^	229.6^a^	0.68	<0.001	0.006	0.089
Total	1,054.5	1,011.3	1,080.9	1,142.0	70.10	0.618	<0.001	0.056
Body weight (kg)								
At birth	35.3	36.1	37.9	37.4	1.74	0.713	-	-
At weaning (56 d)	67.2^ab^	59.1^b^	70.6^ab^	76.6^a^	4.19	0.054	-	-
ADG (g/d)	576^ab^	400^b^	591^ab^	721^a^	75.9	0.045	<0.001	0.014
Gain-to-feed ratio	0.54^a^	0.35^b^	0.53^ab^	0.62^a^	0.051	0.007	<0.001	0.001
Body measures gain (cm/wk)								
Withers-height	1.28	0.99	1.25	1.43	0.153	0.255	0.762	0.216
Hip-width	0.57^ab^	0.48^b^	0.69^ab^	0.77^a^	0.006	0.010	0.015	0.759
Hearth-girth	2.37	1.83	2.43	2.72	0.271	0.163	0.024	0.793

SEM, standard error of the mean; DM, dry matter; ADG, average daily gain.

1) WMP, whole milk powder diluted to 125 g/L solids; 48L, milk replacer with moderate lactose (485 g DM/kg) diluted to 125 g/L solids; 53L, milk replacer with high lactose (533 g DM/kg) diluted to 125 g/L solids; 16TS, 53L milk replacer corrected to 160 g/L total solids by the inclusion of a corrector.

2) LD, liquid diet; A, age; LD×A, interaction between liquid diet and age.

3) Refers to nutrient intake coming from the liquid diet only.

a–d Means within a row with different superscripts are significantly different (p≤0.05).

**Table 3:** Blood parameters of calves fed with different liquid diets

Item	Treatments^1)^				SEM	p-value^2)^		
	
	WMP	48L	53L	16TS		LD	A	LD×A
Glucose (mg/dL)	106.4	101.5	112.6	100.3	4.82	0.299	0.017	0.065
Total protein (g/dL)	5.74	5.32	5.63	5.95	0.180	0.115	<0.001	0.948
Albumin (g/dL)	2.85^a^	2.53^b^	2.63^b^	2.85^a^	0.054	0.002	0.003	0.279

SEM, standard error of the mean; DM, dry matter.

1) WMP, whole milk powder diluted to 125 g/L solids; 48L, milk replacer with moderate lactose (485 g DM/kg) diluted to 125 g/L solids; 53L, milk replacer with high lactose (533 g DM/kg) diluted to 125 g/L solids; 16TS, 53L milk replacer corrected to 160 g/L total solids by the inclusion of a corrector;

2) LD, liquid diet; A, age; LD×A, interaction between liquid diet and age.

a,b Means within a row with different superscripts are significantly different (p≤0.05).

**Table 4:** Health parameters of calves fed with different liquid diets

Item	Treatments^1)^				SEM	p-value^2)^		
	
	WMP	48L	53L	16TS		LD	A	LD×A
Fecal score	1.92	2.05	1.95	1.73	0.163	0.596	<0.001	0.580
Days with diarrhea	13.7	14.6	12.5	9.1	2.48	0.446	-	-
Days medicated	4.1	5.4	3.1	5.8	1.00	0.262	-	-
Days on electrolytes	5.0	3.12	2.7	1.7	1.25	0.319	-	-

SEM, standard error of the mean; DM, dry matter.

1) WMP, whole milk powder diluted to 125 g/L solids; 48L, milk replacer with moderate lactose (485 g DM/kg) diluted to 125 g/L solids; 53L, milk replacer with high lactose (533 g DM/kg) diluted to 125 g/L solids; 16TS, 53L milk replacer corrected to 160 g/L total solids by the inclusion of a corrector.

2) LD, liquid diet; A, age; LD×A, interaction between liquid diet and age.
